# Professional Values in Nursing: A Comparative Study between Brazilian and Albanian Students

**DOI:** 10.1590/0034-7167-2025-0239

**Published:** 2026-10-19

**Authors:** Chennyfer Dobbins Abi Rached, Nertila Podgorica, Nicole Yamada Crescente, Emiljano Pjetri, Gleyciane Santos Vieira, Gerhard Müller, Vera Gjinaj

**Affiliations:** I Universidade de São Paulo. São Paulo, São Paulo, Brazil; II UMIT - Private University for Health Sciences, Medical Informatics and Technology. Hall in Tirol, Austria; III University of Shkodra “Luigj Gurakuqi“. Shkoder, Albania

**Keywords:** Education, Nursing, Nursing Education Research, Students, Nursing, Ethics, Professional, Nursing., Educação em Enfermagem, Pesquisa em Educação em Enfermagem, Estudantes de Enfermagem, Ética Profissional, Enfermagem., Educación en Enfermería, Investigación en Educación de Enfermería, Estudiantes de Enfermería, Ética Profesional, Enfermería.

## Abstract

**Objectives:**

to compare Brazilian and Albanian nursing students’ professional values.

**Methods:**

a descriptive-comparative study conducted at three universities per country (November 2020-July 2021). Data from the Nurses Professional Values Scale Three via electronic survey included 337 Brazilian and 347 Albanian students.

**Results:**

Brazilians scored higher across all domains. Total: 124.93 (SD 11.72; 95%CI 123.64-126.14) in Brazil versus 110.86 (SD 14.35; 95%CI 109.35-112.37) in Albania (p<0.001). Caring: Brazil 46.61 (SD 3.70; 95%CI 46.18-46.97) versus Albania 40.65 (SD 5.44; 95%CI 40.07-41.21). Activism: Brazil 43.78 (SD 5.66; 95%CI 43.16-44.36) versus Albania 38.93 (SD 5.92; 95%CI 38.31-39.56). Professionalism: Brazil 34.55 (SD 3.80; 95%CI 34.13-34.94) versus Albania 31.28 (SD 4.21; 95%CI 30.83-31.72) (all p<0.001).

**Conclusions:**

differences and similarities reflect health policies, delivery systems, and nurses’ roles, and gender associated with higher professional values. Findings inform education and policy in both countries and global nursing practice worldwide.

## RESUMO

**Objetivos:**

comparar os valores profissionais de estudantes de enfermagem brasileiros e albaneses.

**Métodos:**

estudo descritivo-comparativo realizado em três universidades de cada país (novembro de 2020 a julho de 2021). Os dados foram coletados por meio da *Nurses Professional Values Scale Three*, utilizando um questionário eletrônico aplicado a 337 estudantes brasileiros e 347 albaneses.

**Resultados:**

os brasileiros obtiveram pontuações mais altas em todos os domínios. Total: 124,93 (DP 11,72; IC95% 123,64-126,14) no Brasil *versus* 110,86 (DP 14,35; IC95% 109,35-112,37) na Albânia (p<0,001). Cuidado: Brasil 46,61 (DP 3,70; IC95% 46,18-46,97) *versus* Albânia 40,65 (DP 5,44; IC95% 40,07-41,21). Ativismo: Brasil 43,78 (DP 5,66; IC95% 43,16-44,36) *versus* Albânia 38,93 (DP 5,92; IC95% 38,31-39,56). Profissionalismo: Brasil 34,55 (DP 3,80; IC95% 34,13-34,94) *versus* Albânia 31,28 (DP 4,21; IC95% 30,83-31,72) (todos p<0,001).

**Conclusões:**

as diferenças e semelhanças refletem as políticas de saúde, sistemas de prestação de serviços e papéis dos enfermeiros, sendo o gênero associado a valores profissionais mais elevados. Os resultados contribuem para a educação e políticas públicas em ambos os países e para a prática global de enfermagem em todo o mundo.

## RESUMEN

**Objetivos:**

comparar los valores profesionales de estudiantes de enfermería brasileños y albaneses.

**Métodos:**

estudio descriptivo-comparativo realizado en tres universidades por país (noviembre de 2020-julio de 2021). Los datos de la da *Nurses Professional Values Scale Three*, obtenida mediante una encuesta electrónica, incluyeron a 337 estudiantes brasileños y 347 albaneses.

**Resultados:**

los brasileños obtuvieron puntuaciones más altas en todos los dominios. Total: 124,93 (DE 11,72; IC95% 123,64-126,14) en Brasil frente a 110,86 (DE 14,35; IC95% 109,35-112,37) en Albania (p<0,001). Cuidado: Brasil 46,61 (DE 3,70; IC95% 46,18-46,97) frente a Albania 40,65 (DE 5,44; IC95% 40,07-41,21). Activismo: Brasil 43,78 (DE 5,66; IC95% 43,16-44,36) frente a Albania 38,93 (DE 5,92; IC95% 38,31-39,56). Profesionalismo: Brasil 34,55 (DE 3,80; IC95% 34,13-34,94) *versus* Albania 31,28 (DE 4,21; IC95% 30,83-31,72) (p<0,001 para todos).

**Conclusiones:**

las diferencias y similitudes reflejan las políticas sanitarias, los sistemas de prestación de servicios y los roles de las enfermeras, y el género se asocia con mayores valores profesionales. Los hallazgos fundamentan la formación y las políticas en ambos países y en la práctica enfermera global.

## INTRODUCTION

A value is a conviction in an idea’s worth that shapes an individual’s behavior pattern^(1)^. Professional values are accepted standards within a professional group, offering a framework for expectations, norms, and individual and organizational integrity assessment^(2)^. In nursing, professional values such as human dignity, compassion, integrity, and social justice distinguish nursing care from other human interactions^(2,3)^. These values must be cultivated during nursing education as a foundation for practice^(4)^.

Nursing students are expected to internalize the professional values outlined by organizations such as the American Nurses Association^(5,6)^, the Albanian Code of Deontology^(7)^, and the Brazilian Code of Ethics^(8)^, particularly when managing ethical dilemmas. These values are central to undergraduate nursing education in both Albania and Brazil. Students must integrate personal and professional values as they adapt to their evolving roles during training^(9,10)^.

In the context of nursing, professional values are often operationalized through the domains assessed by the Nurses Professional Values Scale-Three (NPVS-3): caring, activism, and professionalism. Caring encompasses attitudes and behaviors that promote human dignity, empathy, and respect in nurse-patient relationships, reflecting the ethical commitment to alleviate suffering and protect life^(2,4,6)^. Activism refers to engagement in advancing public health, influencing health policy, promoting social justice, and contributing to the development of nursing science^(2)^. Professionalism involves adherence to ethical codes, accountability, lifelong learning, and the fulfillment of professional responsibilities, including leadership and evidence-based practice^(2,8)^. These domains represent complementary dimensions of nurses’ professional identity, providing a structured framework to assess how students internalize and prioritize the values that guide ethical and competent practice in diverse healthcare settings.

Culture plays a significant role in shaping professional values, influencing perceptions, decision-making, and behaviors through socialization^(4,11)^. Understanding how cultural and geographical contexts impact professional values is important, as these values are linked to job satisfaction, quality of care, and turnover intention^(12)^. Insight into cultural differences can inform strategies to enhance nursing care globally.

Despite recognition that nurses’ personal values differ across cultures, limited research exists comparing professional values among nursing students. Identifying shared and divergent professional values between Albanian and Brazilian students contributes to a global understanding of nursing identity and supports culturally competent care.

This study aimed to examine and compare Brazilian and Albanian nursing students’ professional values. Considering their distinct healthcare systems and nursing practices, exploring these differences can highlight opportunities for educational improvement and professional development. Cross-national comparisons offer benchmarking possibilities, allowing assessment of professional identity formation and educational outcomes.

Knowledge exchange between countries fosters the advancement of nursing practice and education. Comparative analyses between Brazil and Albania can enhance understanding of healthcare challenges across cultural, political, and economic contexts, promoting mutual learning.

The study’s hypothesis is: are there significant differences in caring, activism, and professionalism values between Brazilian and Albanian nursing students? Given the substantial cultural, political, and healthcare system differences between Brazil and Albania, the comparative approach adopted in this study is not intended to imply direct equivalence between the two contexts. Instead, it aims to explore how distinct socio-economic, educational, and historical trajectories shape the internalization of professional nursing values. This cross-national comparison provides an opportunity to identify context-specific strengths and challenges, enrich the interpretation of results, and inform strategies that may be transferable or adaptable across diverse settings. By explicitly examining countries with contrasting profiles, the study seeks to deepen the global understanding of how professional identity in nursing is influenced by structural and cultural determinants.

## OBJECTIVES

To compare Brazilian and Albanian nursing students’ professional values, aiming to enhance understanding of global nursing professional behavior.

## METHODS

### Ethical aspects

The study was developed in accordance with the Helsinki Declaration, and ethical approval was obtained from the Research Committees of the School of Nursing at the *Universidade de São Paulo* and the University of Shkoder in Albania. Weis and Schank, the authors from Marquette University, gave permission to use the instrument. All participants received information about the research and signed the Informed Consent Form (ICF).

### Study design

This is a cross-sectional quantitative study. Data was collected through an electronic survey between November 2020 and July 2021. The study was conducted in the nursing school of three universities in Brazil and three in Albania.

To prepare the manuscript, STrengthening the Reporting of OBservational studies in Epidemiology recommendations guided by the SQUIRE tool were followed.

### Methodological procedures

A sample size calculation was performed to determine the minimum number of participants required to compare the mean NPVS-3 scores between nursing students from Brazil and Albania. The formula for comparing two independent means was applied, considering a 5% significance level (Zα/2 = 1.96), 80% statistical power (Zβ = 0.84), an estimated standard deviation of 12 points, and a minimum clinically relevant difference of five points.

The standard deviation estimate was based on previous validity studies of the NPVS-3 conducted in Brazil and Albania, which reported similar variability in student populations^(13-15)^. The clinically relevant difference of five points was defined according to prior psychometric research and empirical studies using the NPVS-3, which indicated that this threshold represents a meaningful difference in the degree of value adherence. Although the NPVS-3 does not have established clinical cut-offs, this criterion was adopted to reflect a moderate yet significant difference with potential implications for nursing education.

No statistical adjustment was made for multiple comparisons between the NPVS-3 domains (caring, activism, and professionalism), as the primary outcome was the total NPVS-3 score. Domain-level analyses were considered exploratory and interpreted with caution.

The calculation indicated a minimum of 134 participants per group. To increase the accuracy of estimates and compensate for potential losses, all eligible students from the selected universities were included, resulting in 337 participants in Brazil and 347 in Albania.

The inclusion criteria were designed to encompass all academic semesters, thereby enabling the sample to capture students at various stages of their education, from the initial training phase to more advanced stages of their studies.

The study aimed to assess the professional values of all nursing students enrolled in the selected universities across all academic semesters to capture a broad spectrum of experiences, backgrounds, and stages of professional identity formation. For this reason, no exclusion criteria were defined for participants or institutions, except for the pilot test group, which was excluded from the final analysis. Including all eligible students increased representativeness, minimized selection bias, and enabled a more comprehensive comparison between the Brazilian and Albanian contexts. This approach ensured that the results reflect the diversity inherent to nursing education in each country, thus strengthening the external validity of the findings.

University selection was designed to encompass a diverse range of demographic, socioeconomic, and educational backgrounds, reflecting the diversity within nursing education in both Brazil and Albania. By including institutions that attract students from diverse regions and social contexts, this sample is considered representative of the broader nursing student populations in both countries. This diversity enhances the generalizability of the findings, making them more applicable to the overall nursing student demographic in each country.

In this study, “educational, sociodemographic, and economic diversity” refers to the heterogeneity between participating institutions in geographic location (urban, peri-urban, and rural settings), institutional type (public or private), program orientation (community-health versus hospital-centric curricula), faculty resources, and range of clinical placement opportunities. It also encompasses differences in student demographics, such as age, gender distribution, urban/rural origin, and first-generation status, as well as economic factors, including institutional resourcing, tuition and financial-aid policies, and students’ socioeconomic backgrounds. This multidimensional diversity was intentionally included to ensure that the sample reflected the variety of contexts in which nursing education occurs in Brazil and Albania, thereby strengthening the external validity of the findings.

To ensure demographic, socioeconomic, and educational diversity, the participating universities were intentionally selected to represent different geographic locations (urban, peri-urban, and rural), institutional types (public and private), and curricular orientations (community-based and hospital-centered). Within each institution, course coordinators provided complete lists of eligible students, from which all were invited to participate. Invitations were sent via institutional email with a direct link to the Google Forms^®^ survey, ensuring equal opportunity for access. To minimize potential selection bias inherent to online data collection, coordinators sent follow-up reminders and monitored response rates across semesters and institutions. This procedure helped to maintain balanced representation and reduce the risk of overrepresentation of students with greater internet access or interest in the topic.

### Study design

Course coordinators recruited participants by listing eligible students and their email addresses. Data collection, via Google Forms^®^, included the study invitation, the ICF, and the NPVS-3, and was emailed to students.

The NPVS-3^(2)^ comprises two sections: sociodemographic data (age, gender, academic semester, education level, and nursing career aspects); and assessment of professional values. Developed by Weis and Schank^(2)^, the instrument has demonstrated reliability and validity in studies with nursing students and professionals in Brazil and Albania^(13-15)^. It includes 28 items on a five-point Likert scale (1 = not important to 5 = most important), with total scores ranging from 26 to 140, where higher scores reflect greater adherence to professional values. There is no universally established cut-off score for the NPVS-3 to classify adherence levels as high, moderate, or low. In the present study, the instrument was analyzed as a continuous measure, with higher scores indicating greater adherence to professional nursing values.

The scale is divided into three domains: caring (nurse-patient relationships); activism (public health and nursing science advancement); and professionalism (professional conduct and responsibilities)^(13-15)^. In addition to the total NPVS-3 score, domain-specific scores were calculated for caring, activism, and professionalism by summing the scores of the items corresponding to each domain. Higher scores in each domain indicate greater adherence to the specific set of professional values it represents. Descriptive statistics and group comparisons were performed both for the total score and for each domain separately.

A pilot study with ten students tested the clarity of terms, objectivity of instructions, ICF comprehension, suitability for digital use, form layout, and ease of data entry. No difficulties were reported, and pilot participants were excluded from the final sample.

### Data analysis

Statistical analysis was performed using the Statistical Package for the Social Sciences (SPSS) version 15.0 (SPSS Inc., Chicago, IL, USA). Measures of central tendency included mean, standard deviation, minimum, maximum, and percentages. Group comparisons were conducted using the Wilcoxon-Mann-Whitney and Brunner-Munzel tests, with p<0.05 considered statistically significant. Non-parametric tests were chosen due to the non-normal distribution of data and sample size characteristics. The NPVS-3 scores’ ordinal nature and potential variance inequality justified using these tests over parametric alternatives such as two-way ANOVA, ensuring more accurate comparisons, especially between groups of different sizes.

## RESULTS

Table 1 shows the sociodemographic characteristics of Brazilian (n=337) and Albanian (n=347) nursing students.

**Table 1:** Sample sociodemographic characteristics

Factor	Level	Count	Percentage	Lo.95%CI	Hi.95%CI
University	Brazil	337	49.26	57.03	67.32
	Albania	347	50.74	50.63	58.08
Total sample size		684	100		
Semester in Brazil	1^st^	2	0.60	0.02	2.31
	2^nd^	50	15.02	11.56	19.27
	3^rd^	28	8.41	5.84	11.92
	4^th^	58	17.42	13.71	21.87
	5^th^	21	6.31	4.12	9.49
	6^th^	83	24.92	20.57	29.85
	7^th^	27	8.11	5.59	11.58
	8^th^	58	17.42	13.71	21.87
	9^th^	6	1.80	0.73	3.97
	Missing	4	1.19		
Semester in Albania	1^st^	28	8.06	0.00	1.66
	2^nd^	62	17.86		18.72
	3^rd^	30	8.64	9.35	16.08
	4^th^	59	17.02	11.25	18.43
	5^th^	28	8.06	5.43	10.98
	6^th^	47	13.54	14.14	21.91
	Missing	93	26.82		
Gender	Brazilian female	282	83.68	79.34	87.26
	Albanian female	292	84.14	81.30	89.20
	Brazilian male	55	16.32	12.74	20.66
	Albanian male	27	15.85	13.02	19.44
Age	Mean Age Brazil	20.10±4.27	100.0	45.18	46.05
	Mean Age Albania	19.68±5.80	100.0	42.44	43.62

* *95% Confidence Interval as a reference (The range is calculated for a statistic and not a variable, so this range is for a proportion of studies in each category); Lo.CI - low Confidence Interval; Hi.CI - high Confidence Interval.*

Brazilian nursing students’ mean age was 20.10 years (SD 4.27). Albanian students’ mean age was 19.68 years (SD 5.80). In both countries, students typically begin nursing studies in their early twenties, following high school. However, Brazil’s nursing student population tends to be more socioeconomically diverse, reflecting the country’s broader urbanization and regional disparities.

Female students constituted 83.68% of the Brazilian sample and 84.14% of the Albanian sample. In Brazil, 24.90% of students were in the sixth semester, while in Albania, 13.54% were in their final semester. Nursing remains a predominantly female profession in both countries, although Albania may present slightly higher male representation in urban settings.

Comparing students from the same academic semester is critical for ensuring consistency in academic progress and educational exposure. Students at different stages exhibit varying levels of clinical experience, theoretical knowledge, and professional development. Aligning semester cohorts enhances comparability by controlling for differences related to educational maturity.

Additionally, matching students by semester minimizes external influences linked to the academic journey, such as early-stage adaptation challenges or final-year employment pressures. This methodological control reduces variability in psychosocial stressors that could otherwise confound the results.

Controlling for academic semester also mitigates maturation bias. Over time, students naturally develop greater clinical competence, ethical awareness, and coping mechanisms. Comparing students at similar points in their education limits the influence of these developmental changes, thereby enhancing the internal validity of the study findings.

The comparison between participants from Albania and Brazil revealed statistically significant differences across all assessed dimensions: total NPVS-3; caring; activism; and professionalism. Hypothesis testing indicated p-values below 0.001 for all comparisons, highlighting that the observed differences are unlikely to have occurred by chance. Consistently, Brazilian participants showed higher median scores across all analyzed dimensions. Specifically, analysis of the total NPVS-3 factor using the Brunner-Munzel test indicated a significant difference, with a statistic of 15.60 (df = 679.7; p < 0.001). Similar results were observed for the caring factor, also assessed using the Brunner-Munzel test (statistic = 20.35; df = 672.4; p < 0.001). For the activism and professionalism dimensions, Wilcoxon-Mann-Whitney tests confirmed the differences between the groups, with p < 0.001 (Table 2 and Table 3).

**Table 2:** Descriptive measures by country

Variable	Country	N	Mean	SD	Min.	1^st^Q	Median	3^rd^Q	Max.	Lo.CI	Hi.CI
Total NPVS-3	Albania	347	110.86	4.35	80	99	111	122	140	109.35	112.37
	Brazil	337	124.93	11.72	86	117	128	134	140	123.64	126.14
Caring	Albania	347	40.65	5.44	27	37	41	45	50	40.07	41.21
	Brazil	337	46.61	3.70	25	45	47	49	50	46.18	46.97
Activism	Albania	347	38.93	5.92	27	34	39	44	50	38.31	39.56
	Brazil	337	43.78	5.66	28	40	45	49	50	43.16	44.36
Professionalism	Albania	347	31.28	4.21	18	28	31	34	40	30.83	31.72
	Brazil	337	34.55	3.80	21	32	35	38	40	34.13	34.94

* *95% Confidence Interval regarding proportions (The interval is calculated for a statistic and not for a variable; therefore, this interval is for the statistic proportion of subjects in each category); SD - standard deviation; Min. - minimum; Max. - maximum; 1^st^Q - first quartile; 3^rd^Q - third quartile; Lo.CI - low Confidence Interval; Hi.CI - high Confidence Interval; NPVS-3 - Nurses Professional Values Scale-Three.*

**Table 3:** Hypothesis tests

Factor	Method	Statistic	df	*p* value	
Total NPVS-3	Brunner-Munzel test	15.60	679.7	< 0.001	^***^
Caring	Brunner-Munzel test	20.35	672.4	< 0.001	^***^
Activism	Wilcoxon-Mann-Whitney test	31907.5		< 0.001	^***^
Professionalism	Wilcoxon-Mann-Whitney test	33088.5		< 0.001	^***^

*df - degrees of freedom; NPVS-3 - Nurses Professional Values Scale-Three.*

Brazilian students achieved higher total and domain-specific scores for each construct of the NPVS-3 compared to those from Albania. For Brazilian students, the NPVS-3 total mean score was 124.93 ± 11.72 (p < 0.001), with mean scores of 46.61 ± 3.70 for caring, 43.78 ± 5.66 for activism, and 34.55 ± 3.80 for professionalism. In contrast, Albanian students had a NPVS-3 total mean score of 110.86 ± 14.35 (p < 0.001), with mean scores of 40.65 ± 5.44 for caring, 38.93 ± 5.92 for activism, and 31.28 ± 4.21 for professionalism. The boxplots visually reinforce these differences, showing higher medians for Brazilian participants across all dimensions despite outliers, particularly in the caring domain. These findings suggest that, within the assessed context, individuals from Brazil tend to express higher levels of values and behaviors related to caring, activism, and professionalism than individuals from Albania (Table 2 and Table 3).

Differences in NPVS-3 scores between Brazil and Albania reflect sociocultural, educational, and systemic factors. Cultural norms shape nursing roles: Brazil’s emphasis on collective well-being may enhance scores in advocacy and equality, while Albania’s traditional values may affect the caring dimension^(16-18)^.

Educational differences also influence values. Brazilian curricula emphasize community health, ethics, and social responsibility, aligned with the Brazilian Unified Healthcare System (In Portuguese, *Sistema Único de Saúde* - SUS), whereas Albanian education prioritizes technical skills, potentially lowering advocacy-related scores^(9,16-18)^.

Healthcare system structures further impact value development. Brazil’s universal healthcare promotes public health and equity values, while Albania’s reforming system, focused on efficiency, may reduce emphasis on activism and patient-centered care^(16-19)^.

Professional identity and autonomy vary: Brazil’s regulated practice fosters higher scores in leadership and ethics, while Albania’s hierarchical structure may limit professional responsibility perceptions.

Economic disparities contribute as well. Brazil’s regional inequalities strengthen advocacy values^(20,21)^, whereas Albania’s centralized system fosters pragmatism and technical focus^(17,18)^.

Nursing’s societal status also differs. In Brazil, nursing’s prestige supports higher scores in professional pride and ethics^(22)^, while in Albania, the profession’s more technical perception may lower leadership and self-esteem scores^(23)^.

Ethical and legal frameworks play a role. Brazil’s established nursing codes^(8)^ reinforce ethical standards, while Albania’s evolving regulations may influence NPVS-3 domain emphasis.

Religious and moral beliefs impact values too. Brazil’s religious diversity fosters compassion and dignity^(24)^, whereas Albania’s secular and religious mix shapes different ethical priorities^(23)^.

Finally, female participants showed higher NPVS-3 scores in both countries, consistent with associations between nursing, empathy, and nurturing behaviors (Table 4).

**Table 4:** Descriptive measures by gender in Brazil and Albania

Variable	Gender	N	Mean	SD	Min.	1^st^Q	Median	3^rd^Q	Max.	Lo.95%CI	Hi. 95%CI
**Brazil**											
Caring	Female	282	46.99	3.59	25	46	48	50	50	46.38	47.46
	Male	55	46.14	4.04	33	45	47	49	50	44.55	47.20
Activism	Female	282	44.10	5.66	28	40	46	49	50	43.22	44.91
	Male	55	41.86	5.25	28	39	43	45	50	40.03	43.39
Professionalism	Female	282	34.62	3.71	25	32	35	38	40	34.06	35.16
	Male	55	33.43	3.80	24	31	33	36	40	32.14	34.56
**Albania**											
Caring	Female	292	40.61	4.32	25	40	47	49	50	45.12	46.05
	Male	27	39.86	3.95	33	39	46.5	49	50	44.51	46.86
Activism	Female	292	38.29	5.75	26	40	44	48	50	42.65	43.89
	Male	27	35.25	5.99	25	37.25	42	45.25	50	39.35	42.86
Professionalism	Female	292	31.13	3.92	21	30	34	37	40	33.70	34.54
	Male	27	30.16	3.95	24	30	33	36	40	31.96	34.27

* *95% Confidence Interval regarding proportions (The interval is calculated for a statistic and not for a variable; therefore, this interval is for the statistic proportion of subjects in each category); SD - standard deviation; Min. - minimum; Max. - maximum; 1^st^Q - first quartile; 3^rd^Q - third quartile; Lo.CI - low Confidence Interval; Hi.CI - high Confidence Interval.*

As shown in Table 4, female students presented higher mean scores than male students across the three NPVS-3 domains in both countries.

Female nursing students consistently score higher than male students on the NPVS-3, especially in caring, activism, and professionalism. These differences stem from sociocultural norms linking women to caregiving and empathy, traits aligned with nursing’s core values^(7,8)^.

Historically, nursing has extended women’s familial caregiving roles, strengthening their professional identity, while male students often associate nursing with technical or leadership roles. Societal stereotypes and biases in education may further reinforce these trends^(25,26)^.

Female students typically achieve higher caring scores due to greater encouragement to develop empathy and relational skills^(27,28)^. In activism, their sensitivity to social justice issues enhances their advocacy role, while male students may view advocacy as secondary to clinical duties^(29-31)^.

In relation to professionalism, women show stronger commitment to ethics and patient-centered care, aligning their professional identity more closely with nursing values, while men may experience role conflicts^(9,10,32)^.

Self-selection based on alignment with caregiving values also contributes, as women often enter nursing driven by compassion and advocacy ideals, while men cite diverse motivations such as technology or career stability^(33-35)^.

Educational practices may unintentionally reinforce these gender differences. To foster inclusivity, nursing education must address implicit biases and support diverse motivations for embracing nursing’s core values^(36-38)^.

## DISCUSSION

This study demonstrates that core professional values are prevalent among nursing students from Brazil and Albania, with the highest scores in the caring domain. Brazilian students prioritized items 2, 3, 6, 18, and 20, while Albanian students prioritized 2, 3, 15, 18, and 19. Respect for human dignity and patient safety (items 2 and 3) were highly valued by all students, aligning with national Codes of Ethics that emphasize respect for life and human rights in both countries^(7,8)^.

Gender emerged in this study as a central determinant of professional values in nursing students, consistent with existing literature indicating that nursing remains a profession strongly associated with socially constructed notions of femininity, empathy, and care^(37-41)^. Female students’ higher scores in the caring, activism, and professionalism domains may reflect both self-selection into the profession based on alignment with caregiving ideals^(33-35)^ and the reinforcement of these attributes through formal and informal aspects of nursing education^(36-38)^. However, these patterns cannot be fully understood without considering intersectional factors, as gender does not operate in isolation. An intersectional approach recognizes how overlapping social markers-such as gender, socioeconomic background, and geographical origin mutually shape opportunities, perceptions, and professional identity development^(42-46)^. In our sample, the recruitment of students from different universities, with varied geographic locations and institutional profiles, introduces heterogeneity that interacts with gender in complex ways, potentially amplifying or mitigating certain professional values.

Moreover, the inclusion of students from both Brazil and Albania enables an exploration of how gender intersects with territory-specific cultural norms, healthcare structures, and educational systems to shape professional values^(16-19,45)^. Territorial context whether urban, peri-urban, or rural can influence not only access to diverse clinical experiences but also exposure to different patient populations and health inequities, thereby affecting students’ ethical perspectives and activism orientation^(19,20,23)^. In Brazil, the strong emphasis on community health and social justice in nursing curricula, aligned with the SUS principles^(16,19)^, may interact with gendered expectations to reinforce activism and advocacy roles. In Albania, where nursing education remains more technically oriented within a physician-centered healthcare model^(18,45)^, these intersections may manifest differently, potentially limiting the development of activism-related values. Considering these intersectional dynamics strengthens the rationale for a cross-national comparison, as it moves beyond a binary gender analysis and situates the findings within broader socio-cultural, economic, and territorial contexts, thereby aligning with the study’s aim of contributing to a global understanding of nursing professional behavior.

Professional values in nursing, shaped historically by political systems and institutions such as the International Council of Nurses^(6)^, encompass responsiveness, compassion, trust, and integrity. High scores on altruism-related values were consistent with previous findings^(39-41)^, reflecting a strong patient-centered ethos among students.

Gender influences professional values, as nursing is traditionally associated with female attributes such as caring and nurturing^(37,38)^. Studies show that femininity correlates with caring behavior^(42)^ and emotional intelligence^(43)^, supporting findings that female students in both countries achieved higher values scores, particularly in the caring domain^(44)^.

Differences in healthcare systems contribute to the variance in activism values. In Brazil, the SUS emphasizes universal, equitable healthcare^(19)^, fostering a culture of social justice and activism among nursing students. Brazilian nurses’ involvement in public health and policy-making historically stems from strong social movements^(19)^. In contrast, Albania’s transition from a communist to a democratic healthcare system^(45)^ has resulted in a physician-centered, less activist healthcare culture.

Nurses in Albania focus more on clinical skills than systemic advocacy^(18,45)^. The persistence of hierarchical structures and resource limitations also constrains the development of activism. Promoting patient-centered care and democratizing healthcare interactions are essential to enhancing professional values among Albanian nursing students^(45)^.

Academic progression influences professional values acquisition. Higher semester students generally report stronger professional identities^(13,14)^. Clinical exposure and mentorship from experienced educators promote critical thinking, ethical decision-making, and professional socialization^(46,47)^.

Activism, the second-highest valued domain, reflects students’ commitment to public health promotion^(2)^. Public healthcare systems in both countries integrate social justice principles into nursing education^(16,20,23)^. However, Brazilian students’ higher activism scores may be due to greater involvement in health policy processes^(16,20)^, highlighting the role of social participation in strengthening activism.

Caring behavior correlates with critical thinking and social justice promotion, both influenced by gender expectations^(42)^. This intersection underscores the need to address gender norms in nursing education to enhance professional identity formation.

Professionalism received the lowest scores among the constructs but remained positive. Higher engagement with scientific research enhances students’ perception of evidence-based practice^(48)^. Gender bias may hinder the professional self-concept, with male students facing challenges reconciling caring roles with professional advancement^(42,43)^. Nursing education must critically assess and counteract gender stereotypes to foster a stronger professional identity.

### Study limitations

This study had some limitations, including the use of self-reported data, which may introduce response biases. Additionally, the cross-sectional design limits the ability to infer causality, and the study population was limited to specific universities, which may affect external validity. Moreover, cultural factors that may have influenced the responses were not fully assessed.

Although the sample captured substantial educational, sociodemographic, and economic diversity, these factors were not used as stratification variables or covariates in the statistical analyses. As such, potential within-country differences linked to institutional type, geographic location, or student socioeconomic background could not be disentangled. Future studies could incorporate stratified sampling or multivariate models to explore how these contextual variables interact with the development of professional nursing values.

The findings of this study allow us to reflect that the lack of development of nursing students’ professional values during training compromises professionals’ initial performance. Hence, it will have an instrument to measure professional values during the auxiliary exercise of teaching staff in the analysis and implementation of teaching-learning strategies.

In addition to the limitations already noted, it is important to highlight that undergraduate nursing programs in the two countries differ in duration, nine semesters in Brazil and six in Albania, which implies variations in students’ exposure to theoretical content, clinical practice, and ethical training. This discrepancy may influence the degree of internalization of professional values, as students in more advanced stages of their studies tend to demonstrate greater academic and professional maturity. Furthermore, in the Albanian sample, there was a high proportion of missing data for the “semester” variable (26.82%) resulting from non-completion of this field in the electronic questionnaire. These factors limit the direct comparability between the samples and should be considered when interpreting the results, as they may have contributed to the differences observed in NPVS-3 scores.

### Contributions to nursing, health, or public policy

This study highlights the need to strengthen professional values, particularly activism and professionalism, in nursing education in Albania and Brazil, with tailored strategies for each context.

In Albania, curricula should expand community health exposure and offer practical experiences in underserved areas to reinforce advocacy and social justice. Greater emphasis on ethics, leadership, and autonomy is essential, alongside international exchanges to promote professional identity development.

In Brazil, where activism is strong, education should further deepen professionalism by emphasizing accountability, leadership, and ethical standards across nursing specialties. Formal training in healthcare policy advocacy and interdisciplinary education with other health fields are recommended. Both countries should also implement continuing education programs focused on ethics, leadership, and advocacy to sustain professional values throughout nurses’ careers. By enhancing these areas, nursing education can better prepare students to be skilled clinicians, ethical leaders, and healthcare advocates.

## CONCLUSIONS

This study shows that Brazilian nursing students exhibit higher NPVS-3 scores across caring, activism, and professionalism compared to Albanian students, especially in caring and activism. These results highlight the influence of healthcare systems, policies, education, and societal roles on nursing professional values. Future research should explore cultural factors and apply longitudinal designs.

Professional identity evolves with changing work contexts. Brazilian students’ higher scores suggest stronger value internalization, likely influenced by curricula, research involvement, and nurses’ active roles in public policy.

To strengthen professional values, both countries should incorporate reflective practices into education, using clinical experiences, ethical dilemmas, and case-based learning adapted to local contexts. Mentorship programs, research engagement, and ethical decision-making exercises can further reinforce professional identity. In Albania, expanding exposure to community health, ethics, and autonomy could enhance students’ professional identity. In Brazil, advancing specialized advocacy training, leadership development, and interdisciplinary collaboration is recommended.

Brazilian students’ higher activism scores may reflect greater public policy participation, while higher professionalism scores align with research involvement. Embedding civic engagement and scientific inquiry into curricula is vital to foster ethical leadership.

In conclusion, while both groups prioritize patient care, Brazilian students show stronger alignment with activism and professionalism. Educational strategies focusing on reflection, mentorship, and research are essential to cultivating ethical and leadership skills in future nurses.

## Data Availability

The research data are available in a repository: https://data.mendeley.com/datasets/vfsfbn9mwr/1.
